# A Database-driven Decision Support System: Customized Mortality Prediction

**DOI:** 10.3390/jpm2040138

**Published:** 2012-09-27

**Authors:** Leo Anthony Celi, Sean Galvin, Guido Davidzon, Joon Lee, Daniel Scott, Roger Mark

**Affiliations:** 1 Laboratory of Computational Physiology, Harvard-MIT Division of Health Sciences and Technology, 77 Massachusetts Avenue, E25-505, Cambridge, MA 02139, USA; E-Mails: joonlee@mit.edu (J.L.); djscott@mit.edu (D.S.); rgmark@mit.edu (R.M.); 2 Department of Cardiac Surgery, Dunedin Hospital, 201 Great King Street, Dunedin 9054, New Zealand; E-Mail: galse@me.com; 3 Department of Radiology, Stanford Hospital, 300 Pasteur Drive, Stanford, CA 94305, USA; E-Mail: davidzon@stanford.edu

**Keywords:** decision support, intensive care, clinical database, MIMIC, informatics

## Abstract

We hypothesize that local customized modeling will provide more accurate mortality prediction than the current standard approach using existing scoring systems. Mortality prediction models were developed for two subsets of patients in Multi-parameter Intelligent Monitoring for Intensive Care (MIMIC), a public de-identified ICU database, and for the subset of patients >80 years old in a cardiac surgical patient registry. Logistic regression (LR), Bayesian network (BN) and artificial neural network (ANN) were employed. The best-fitted models were tested on the remaining unseen data and compared to either the Simplified Acute Physiology Score (SAPS) for the ICU patients, or the EuroSCORE for the cardiac surgery patients. Local customized mortality prediction models performed better as compared to the corresponding current standard severity scoring system for all three subsets of patients: patients with acute kidney injury (AUC = 0.875 for ANN, *vs.* SAPS, AUC = 0.642), patients with subarachnoid hemorrhage (AUC = 0.958 for BN, *vs.* SAPS, AUC = 0.84), and elderly patients undergoing open heart surgery (AUC = 0.94 for ANN, *vs.* EuroSCORE, AUC = 0.648). Rather than developing models with good external validity by including a heterogeneous patient population, an alternative approach would be to build models for specific patient subsets using one’s local database.

## 1. Introduction

Medicine today is fundamentally the application of clinical guidelines and practices that have been proven effective in clinical trials. While these standard practices may be effective in the aggregate for populations of patients, all clinical practices are, in the end, delivered to individual patients, not populations. The prospect of genomic-based personalized medicine has seemed just over the horizon for years. We argue that personalized medicine will happen sooner then we expect, but genomics will only be part of the change. The rest will come from large-scale analysis of detailed clinical data. Clinical decision support tools that arise from “big data analytics” will complement and may even replace clinical guidelines as data become more available.

In a previous paper, we described the framework of a dynamic decision support tool based on empiric data, a departure from the typical built-in expert systems derived from published large, usually multi-center, interventional and observational studies [1]. Even though prospective randomized controlled trials and systematic reviews have become gold standard in establishing the link between science and medical practice, important insights continue to arise from routine patient care as documented in a clinical database. In this approach, probabilistic modeling is performed on patient subsets from one’s own institution rather than heterogeneous patient populations from different centers, trading off generalizability for accuracy. We coined the term Collective Experience for this approach, as information is drawn from the experience of various clinicians as stored in the electronic medical record system. In that proof-of-concept paper, we demonstrated that mortality prediction using logistic regression models built on ICU patients who developed acute kidney injury (AKI) performed better than the Simplified Acute Physiology Score (SAPS) on an unseen test set. 

This study aims to test this hypothesis on another group of ICU patients—those presenting with subarachnoid hemorrhage (SAH)—from the same database. We also apply this approach to a different database from another country and compare mortality prediction models for elderly patients (>80 years old) undergoing cardiac surgery against the current standard EuroSCORE. In addition, we explore the use of automated feature selection to identify model variables and different machine learning algorithms for model-building.

## 2. Experimental

### 2.1. MIMIC Database, Beth Israel Deaconess Medical Center (BIDMC), Boston, MA, USA

The Laboratory of Computational Physiology at Massachusetts Institute of Technology (MIT) developed and maintains the Multi-parameter Intelligent Monitoring for Intensive Care (MIMIC) database, consisting of ICU patients admitted to the BIDMC since 2003 that has been de-identified by removal of all Protected Health Information [2]. BIDMC is a 621-bed teaching hospital of the Harvard Medical School with 77 ICU beds. An Institutional Review Board (IRB) approval was obtained for the development, maintenance and public use of the database. 

Using the MIMIC database, we identified patients who had an ICD-9 diagnosis of (1) acute kidney injury (584.9) and (2) subarachnoid hemorrhage (430, 852). The outcome of interest is survival to hospital discharge. The covariates that were evaluated included demographic factors (age and sex), SAPS, and physiologic variables measured during the first three days in the ICU for the patients with AKI, and for the first day for those admitted with SAH. We suspected that the model for the patients with AKI will be more complex and would require evolution of the physiologic variables. We obtained the minimum, maximum, standard deviation and average value of the majority of the physiologic variables. 

The SAPS of each patient was converted to predicted mortality using the following formula:

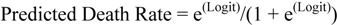

where Logit = 7.7631 + 0.0737*SAPS + 0.9971*ln(SAPS + 1) [3].

The predicted death rate was compared against the true outcome, and an Area under the Receiver Operating Characteristic Curve (AUC) and Hosmer-Lemeshow (HL) Goodness-of-Fit test decile were calculated. 

### 2.2. Registry of Cardiac Surgery Patients in Dunedin Hospital, University of Otago, New Zealand

Dunedin Hospital is a 350-bed teaching hospital of the University of Otago Medical School. It provides tertiary services (including Cardiothoracic and Neurosurgery) to the lower South Island of New Zealand. The Department of Cardiothoracic Surgery is a referral unit providing services to the provinces of Otago, Southland and parts of south Canterbury, an approximate catchment area of 280,000 people, and performs on average 300 open heart surgeries per year. Unlike MIMIC, which archives all the data collected as they are captured throughout the process of care, the registry contains only selected pre-defined variables entered by personnel with domain expertise. The registry does not have high-resolution time series physiologic data unlike MIMIC, but only has discrete pre-operative, intra-operative and post-operative variables. For this paper, a retrospective review of patients over 80 years of age having publicly funded cardiac surgery from January 1995 until February 2006 was undertaken. The predicted death risk, given by the logistic EuroSCORE, was compared against the true outcome, and the AUC and HL decile were likewise calculated. 

**Figure 1:**
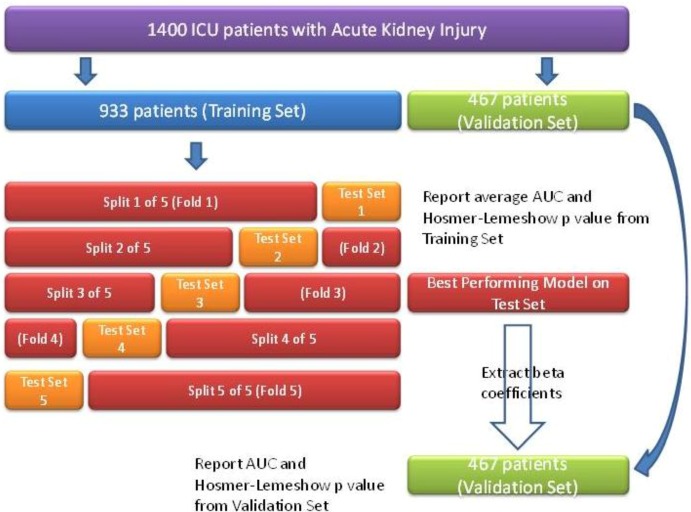
Cross-validation method for ICU patients admitted with acute kidney injury (AKI). AUC = Area under the Receiver Operating Characteristic Curve.

For all three patient cohorts, the correlation-based feature subset selection was employed for variable selection. Correlation-based feature subset (CFS) selection assesses the predictive ability of each attribute individually and the degree of redundancy among them, preferring sets of attributes that are highly correlated with the outcome but have low inter-correlation [4]. The patient cohorts were divided into a training and test data with a 70:30 split. The test set was not used to build any of the models. Using Weka (version 3.5.7; University of Waikato, Hamilton, New Zealand) and R software (R version 2.7.2; The R Foundation for Statistical Computing, Auckland, New Zealand), multivariate models were developed on the variables selected by the correlation-based feature subset algorithm. Five-fold cross-validation was performed on the training set and the best-fitted model was evaluated on the test set (Figure 1). Logistic regression, Bayesian network and artificial neural network were employed. Two sets of AUC were obtained for each model. The first is the average of the five values obtained from each of the cross-validation run. The second was obtained by evaluating the model that performed the best on the training set on the test data, in order to eliminate a learning bias. Only the second set of AUC is reported. The best fitted logistic regression models are presented in detail, and the Hosmer-Lemeshow *p* values are presented for goodness-of-fit. (As a reminder to the readers, the null hypothesis for the Hosmer-Lemeshow statistic is that the model is perfectly calibrated. When the p value less than 0.05, the null hypothesis is rejected, suggesting poor calibration.) We then compared the performance of the best-fitted models on the test data against the performance of SAPS or logistic EuroSCORE. 

## 3. Results and Discussion

### 3.1. Results

#### 3.1.1. ICU Patients with AKI

There were a total of 1,400 patients with an ICD-9 diagnosis of AKI who survived at least 72 h in the ICU. Of these, 970 survived and 430 died in the hospital (30.7% mortality). These were divided into a training set (979 patients) and test set (421 patients). The difference between the mortality rate of the training set (31.9%) and test set (28.2%) is not statistically significant (*p* > 0.05). The difference in the distributions of the variables in the training and test sets is also not statistically significant (data not shown). 

The AUC of SAPS on the test data was 0.6419 and the Hosmer-Lemeshow decile was 2941.64 (*p* = 0). This is the gold standard against which the performance of the multivariate models is compared.

Of the 118 variables considered for this patient cohort, the correlation-based feature subset algorithm selected 21 physiologic variables from the first 3 days for modeling. Below is the performance of three machine-learning algorithms in predicting hospital mortality among patients with AKI using the variables selected by the CFS Subset Evaluator algorithm (Table 1), and the best fitted logistic regression model (Table 2).

**Table 1:** Performance of customized mortality predictions models for ICU patients who developed acute kidney injury (AKI).

	Accuracy	Mean Absolute Error	Area under the ROC Curve
Logistic Regression	72.9%	0.367	0.738
Bayesian Network	73.2%	0.306	0.761
Artificial Neural Network	81.9%	0.227	0.875

**Table 2:** Best fitted logistic regression model for ICU patients who developed AKI.

	Estimate	Standard Error	z value	Pr (>|z|)
Age	5.54e−03	2.30e−03	2.41	0.02
Maximum serum bilirubin (Day 2)	4.58e−02	1.46e−01	0.31	0.75
Maximum serum bilirubin (Day 3)	1.66e−02	1.42e−01	0.12	0.91
Minimum heart rate (Day 2)	3.64e−03	5.44e−03	0.67	0.50
Average systolic blood pressure (Day 1)	−8.61e−03	5.71e−03	−1.51	0.13
Minimum systolic blood pressure (Day 2)	−8.31e−04	6.25e−03	−0.13	0.89
Minimum systolic blood pressure (Day 3)	−2.18e−02	7.42e−03	−2.94	0.003
Average systolic blood pressure (Day 3)	6.46e−03	7.71e−03	0.84	0.40
Maximum respiratory rate (Day 3)	1.58e−02	1.14e−02	1.38	0.17
Standard deviation of the hematocrit (Day 2)	1.05e−01	5.05e−02	2.08	0.04
Minimum White Blood Cell count (Day 1)	−1.19e−03	1.98e−02	−0.06	0.95
Minimum White Blood Cell count (Day 2)	−7.07e−02	8.66e−02	−0.82	0.41
Average White Blood Cell count (Day 2)	6.50e−02	8.59e−02	0.76	0.45
Minimum White Blood Cell count (Day 3)	3.36e−02	2.28e−02	1.47	0.14
Maximum BUN (Day 2)	−1.66e−02	8.29e−03	−2.00	0.05
Maximum BUN (Day 3)	2.98e−02	8.39e−03	3.56	0.0004
Glasgow coma score (Day 1)	−4.42e−02	1.71e−02	−2.59	0.01
Maximum serum bicarbonate (Day 1)	6.20e−03	1.83e−02	0.34	0.73
Urine Output (Day 1)	−1.20e−04	8.44e−05	−1.43	0.15
Urine Output (Day 2)	−6.60e−05	6.75e−05	−0.98	0.33
Urine Output (Day 3)	−1.10e−04	7.44e−05	−1.48	0.14

Note: Hosmer-Lemeshow statistic = 6.472 (*p* = 0.594).

#### 3.1.2. ICU Patients with SAH

Since 2003, there were 223 individual hospitalizations at BIDMC ICU with a discharge diagnosis of subarachnoid hemorrhage. Fifty-seven patients died (25.6%) and 166 patients (74.4%) were discharged alive. These were divided into a training set (150 patients) and test set (73 patients). The difference between the mortality rate of the training set and test set is not statistically significant (*p* > 0.05). The difference in the distributions of the variables in the training and test sets is also not statistically significant (data not shown). 

The AUC of SAPS on the test data was 0.84 and the Hosmer-Lemeshow decile was 887.95 (*p* < 0.001). This is the gold standard against which the performance of the multivariate models is compared.

Of the 53 variables considered for this patient cohort, the correlation-based feature subset algorithm selected 13 physiologic variables from the first 24 h for modeling. Below is the performance of three machine-learning algorithms in predicting hospital mortality among patients with SAH using variables selected by the correlation-based feature subset algorithm (Table 3), and the best fitted logistic regression model (Table 4). 

**Table 3:** Performance of customized mortality predictions models for ICU patients who presented with subarachnoid hemorrhage (SAH).

	Accuracy	Mean Absolute Error	Area under the ROC Curve
Logistic Regression	89.0%	0.158	0.945
Bayesian Network	87.7%	0.127	0.958
Artificial Neural Network	83.6%	0.168	0.868

**Table 4:** Best fitted logistic regression model for ICU patients who presented with SAH.

	Estimate	Standard Error	z value	Pr (>|z|)
Age	0.05	0.02	2.64	0.008
Average serum glucose	0.02	0.01	2.50	0.01
Maximum White Blood Cell count	0.01	0.05	0.10	0.92
Standard deviation of the serum glucose	0.13	0.32	0.41	0.68
Average prothrombin time INR	3.20	1.56	2.05	0.04
Minimum Glasgow coma score	−0.01	0.17	−0.06	0.95
Maximum Glasgow coma score	0.24	0.22	1.12	0.26
Average Glasgow coma score	−0.60	0.33	−1.80	0.07
Minimum systolic blood pressure	−0.02	0.02	−0.94	0.34
Minimum serum sodium	0.03	0.32	0.10	0.92
Average serum sodium	−0.03	0.32	−0.10	0.92
Standard deviation of the serum sodium	0.02	0.44	0.04	0.97

Note: Hosmer-Lemeshow statistic = 7.196 (*p* = 0.516).

#### 3.1.3. Elderly Patients Who Underwent Open Heart Surgery

Among a total of 3,261 patients who underwent heart surgery during the study period, 165 (5.1%) were aged 80 years or more (range 80–94.6, mean 82.3 years). The majority, 96 (59.6%) underwent non-elective surgery. Overall, the in-hospital mortality was 7.4% (3.4% Coronary Artery Bypass Graft or CABG, 9.1% Aortic Valve Replacement or AVR, 10.2% CABG + Valve). For this patient cohort, the logistic EuroSCORE had an AUC of 0.648 and an HL decile of −181.8 (*p* = 1.0).

Of the 41 pre-, post- and operative variables considered for this patient cohort, the correlation-based feature subset algorithm selected six variables for modeling. Below is the performance of three machine-learning algorithms in predicting hospital mortality among the elderly cardiac surgery patients using the variables selected by the correlation-based feature subset algorithm (Table 5), and the best fitted logistic regression model (Table 6).

**Table 5:** Performance of customized mortality predictions models for elderly patients who underwent cardiac surgery.

	Accuracy	Mean Absolute Error	Area under the ROC Curve
Logistic Regression	80.0%	0.201	0.854
Bayesian Network	96.4%	0.129	0.931
Artificial Neural Network	96.4%	0.045	0.941

**Table 6:** Best fitted logistic regression model for elderly patients who underwent cardiac surgery.

	Estimate	Standard Error	z value	Pr (>|z|)
Ejection fraction	1.11	1.01	1.10	0.27
Use of an intra-aortic balloon pump	1.61	1.67	0.97	0.33
Chest Reopening	3.14	1.38	2.28	0.02
Development of atrial fibrillation	18.68	2.46	−0.01	0.99
Development of a post-operative infection	0.77	1.19	−0.65	0.52

Note: Hosmer-Lemeshow statistic = 5.671 (*p* = 0.684).

### 3.2. Discussion

Using a clinical database, we previously demonstrated accurate prediction of fluid requirement of ICU patients who are receiving vasopressor agents using the physiologic variables during the previous 24 h in the ICU [5]. Subsequently, we demonstrated improved mortality prediction among ICU patients who developed AKI by building models on this subset of patients [1]. In this paper, we applied the approach to another subset of patients (ICU patients who presented with SAH) from MIMIC, and to a database from another country (elderly patients who underwent cardiac surgery in Dunedin Hospital, New Zealand). In addition, instead of limiting the variables to those that have been validated to be predictive of mortality, we used an automated feature selection to a large group of candidate variables. Finally, we employed three machine learning algorithms—logistic regression, Bayesian network and artificial neural network—to build our models. 

ICU patients who develop AKI are one subset of patients where severity scoring systems have consistently performed poorly. The largest worldwide multi-center prospective study found that the observed mortality among these patients was substantially greater than predicted by SAPS II (60.3% *vs*. 45.6%, *p* < 0.001) [6]. In another UK-wide study of ICU patients who develop AKI, the APACHE II score under-predicted the number of deaths [7]. In this study, the null hypothesis of perfect calibration was strongly rejected (*p* < 0.001) by both the Hosmer-Lemeshow and Cox’s calibration regression.

SAH is a neurological emergency caused by bleeding into the subarachnoid space. Despite advances in neurocritical monitoring and treatment options, population-based studies reported that SAH death rate is about 50% [8]. Numerous systems are reported for grading the clinical condition of patients following SAH, including the Hunt and Hess Scale, Fisher Scale, Glasgow Coma Score (GCS), and World Federation of Neurological Surgeons Scale. But there are few validation studies of these scales and furthermore, there are no prospective controlled comparison studies [9]. For this reason, the use of any particular SAH grading scale is largely a matter of individual or institutional preference. 

For patients undergoing cardiac surgery, the EuroSCORE is the most frequently used risk algorithm [10]. It was created primarily to allow patient grouping for the total spectrum of cardiac surgery. In the original EuroSCORE model, only 10% of the patients examined were >75 years of age. It is generally accepted that the EuroSCORE places significant weight on age as a surgical risk factor, and as a result, overestimates the mortality in elderly patients undergoing cardiac surgery [11,12]. This small proportion of geriatric patients included in the training patient cohort is a weakness of all current cardiac surgical scoring systems. 

The AUC and Hosmer-Lemeshow p value of SAPS among the MIMIC II patients with AKI that we obtained (AUC = 0.6419, Hosmer-Lemeshow *p* = 0) are consistent with the performance of SAPS in predicting mortality among ICU patients in the US (AUC = 0.67, *p* = 0.05) [13]. In a UK study, APACHE II, another severity scoring system looking at physiologic variables during the first 24 h in the ICU, also had poor calibration (Hosmer-Lemeshow *p* < 0.001) when used to predict death among patients with AKI [7]. Although SAPS performed better among patients in the MIMIC II database presenting with SAH in terms of AUC (AUC = 0.84), its calibration in this patient cohort is very poor (HL *p* < 0.001). As expected, based on previous studies, the EuroSCORE did not discriminate well between survivors and non-survivors among elderly patients who underwent open heart surgery in Dunedin Hospital during the study period (AUC = 0.648). This poor performance of current predictive models when applied to (1) regions different from where the model was built and (2) specific subsets of ICU patients is the main impetus for this research. 

Three machine learning algorithms were employed to build the mortality prediction models. Regression has been the methodology-of-choice in the field of medicine. Most clinicians have some level of familiarity with the concepts behind regression, and the output is relatively easy to understand from a clinical standpoint. However, regression has a number of limitations, including its major assumptions that the predictors are independent and identically distributed (iid), and that the outcome of interest is a function of some linear combination of the variables. The use of quadratic effects and interaction terms allows more complex, but not necessarily better, hypotheses or models. Bayesian or belief network develops models based on conditional probability distribution. The main concept behind the methodology (Is variable A dependent on variable B, or is variable B dependent on variable A, or are variables A and B independent based on the probability distribution?) is not difficult to comprehend. Similar to regression, the output usually makes sense to a clinician. However, conversion of the threshold Bayes factor, which sets the strength of the dependencies between the variables, to the widely-accepted p value has not been well-established. For this reason, the clinical significance of the arcs or dependencies in the network might be more difficult to ascertain. Finally, artificial neural network is a multi-layer perceptron. A perceptron is a linear classifier that defines the hyperplane that separates the data points according to the outcome of interest. Like the Bayesian network, it makes no assumption that the features are iid and complex hypotheses can be explored. However, the output, which consists of nodes and the weights of the variables in each node, is undecipherable to a clinician, making this methodology relatively unpopular in medicine.

For all three patient subsets, the AUCs of the local customized models were significantly higher than those of the gold standards, *i.e.*, SAPS for the ICU patients and EuroSCORE for the cardiac surgery patients. The calibration either improved if the gold standard was poorly calibrated (SAPS), or preserved if the gold standard was well-calibrated (EuroSCORE). For the ICU patients who developed AKI, artificial neural network had the highest AUC. The logistic regression and Bayesian network performed the best for the ICU patients who presented with SAH. Finally, Bayesian network and artificial neural network had the highest AUC for elderly patients who underwent cardiac surgery. The models to predict mortality among elderly patients who underwent cardiac surgery in Dunedin Hospital performed surprisingly well despite a much smaller set of low-resolution data available as candidate variables. 

The gold standard in evidence-based medicine is a well-designed, well-executed multi-center prospective randomized controlled trial. Even when such trials are performed and subsequently published, they very rarely, if ever, provide clear evidence upon which to base the management of an individual patient. Patient prognostication is no exception. There is an abundance of literature on risk assessment performed prospectively. However, patients enrolled in prospective randomized controlled trials are heterogeneous, and conclusions are valid for the “average” patient. In addition, these trials are executed in very strictly monitored, and thus artificial, conditions, and often, findings in these studies do not translate to the real world ICU. It is difficult to predict whether an individual patient is likely to behave like the “average” patient in the multi-center prospective randomized trial. Hence, day-to-day clinical decisions are still based mostly on personal experience, experiences shared with colleagues, and consideration of reported data if they exist. 

Data mining may provide an additional tool for decision support. The main objective of this project is to determine whether predictive models built on patient subsets yield more accurate predictions than traditional one-model-fits-all approach. As more ICUs switch to a paperless system, local ICU database become available for building models. Rather than developing models with good external validity by including a heterogeneous patient population from various centers across countries as has been traditionally done, an alternative approach would be to build models for specific patient subsets using one’s own local or regional database. 

## 4. Conclusions

There are numerous severity scoring systems that are available in the ICU. Although initially designed for mortality prediction, they universally lack clinically acceptable accuracy at an individual patient level [14]. These systems perform relatively well in predicting how many patients will die in an ICU when the individual patient risks are calculated and averaged for that ICU. However, although the prognostic accuracy of the scoring system for an entire ICU population is good, its prognostic accuracy at different levels of risk, or its calibration, is poor. In addition, the performance of these predictive models is always better on entire ICU populations than on specific subsets of patients.

As more hospitals switch to a paperless system, local clinical database become available for building models. Rather than developing models with good external validity by including a heterogeneous patient population from various centers as has been traditionally done, an alternative approach would be to build models for specific patient subsets using one’s own local database.

However, even if we come up with a mortality prediction model based on local institutional empiric data that has excellent sensitivity and specificity, the question remains whether clinicians will embrace this approach. Will we be able to convince them that information from a very large cohort of patients whose clinical course is stored in an electronic database might be more reliable than a composite of the patients they have encountered in the past whose clinical course may be imperfectly stored in their memory? Will we be able to convince them that their clinical intuition on an individual patient might be enhanced by the experience of numerous clinicians who have taken care of clinically similar patients? Impact studies are necessary to evaluate whether this approach will influence clinician behavior and improve patient outcomes.
